# Development of a cost-effective diagnostic algorithm incorporating transcription factor immunohistochemistry in the evaluation of pituitary tumours

**DOI:** 10.1007/s11102-022-01284-2

**Published:** 2022-10-22

**Authors:** N. F. Lenders, J. Chui, J. Low, W. J. Inder, P. E. Earls, A. I. McCormack

**Affiliations:** 1 Department of Endocrinology, St Vincent’s Hospital, Sydney, NSW Australia; 2 Garvan Institute of Medical Research, Sydney, NSW Australia; 3 St Vincent’s Clinical School, University of New South Wales, Sydney, NSW Australia; 4 Department of Anatomical Pathology and Cytopathology, St Vincent’s Pathology, Sydney, NSW Australia; 5 Department of Diabetes and Endocrinology, Princess Alexandra Hospital, Brisbane, QLD Australia; 6 Faculty of Medicine, The University of Queensland, Brisbane, QLD Australia

**Keywords:** Pituitary, Tumour, Transcription factors, Algorithm

## Abstract

**Purpose:**

To determine the utility of the 2022 WHO Classification of pituitary tumours in routine clinical practice and to develop an optimal diagnostic algorithm for evaluation of tumour type in a real-world setting.

**Methods:**

Retrospective evaluation of pituitary tumour immunohistochemistry (IHC), operatively managed at St Vincent’s Hospital Sydney, between 2019 and 2021. Routine IHC comprised evaluation of transcription factors [steroidogenic factor 1 (SF1), T-box transcription factor 19 (TPIT) and pituitary-specific positive transcription factor (PIT1)] and anterior pituitary hormones. Three tiered algorithms were tested, in which hormone IHC was performed selectively based on the initial transcription factor results. These were applied retrospectively and compared with current practice ‘gold standard’ comprising all transcription factor and hormone IHC. Diagnostic accuracy and cost were evaluated for each.

**Results:**

There were 113 tumours included in the analysis. All three algorithms resulted in 100% concordance with the ‘gold standard’ in the characterisation of tumour lineage. While all three were associated with relative cost reduction, Algorithm #3, which omitted hormone IHC in the setting of positive SF1 or TPIT and performed IHC for growth hormone, prolactin and thyroid stimulating hormone only in the setting of PIT1 positivity, was the most cost-efficient. Additionally, there were 12/113 tumours with no distinct cell lineage.

**Conclusion:**

A diagnostic algorithm omitting hormone IHC except in cases of PIT1 positivity is an accurate and cost-effective approach to diagnose the type of pituitary tumour. A significant subgroup of pituitary tumours with no distinct cell lineage, frequently plurihormonal, remains difficult to classify with the new WHO criteria and requires further evaluation.

## Background

**Fig. 1:**
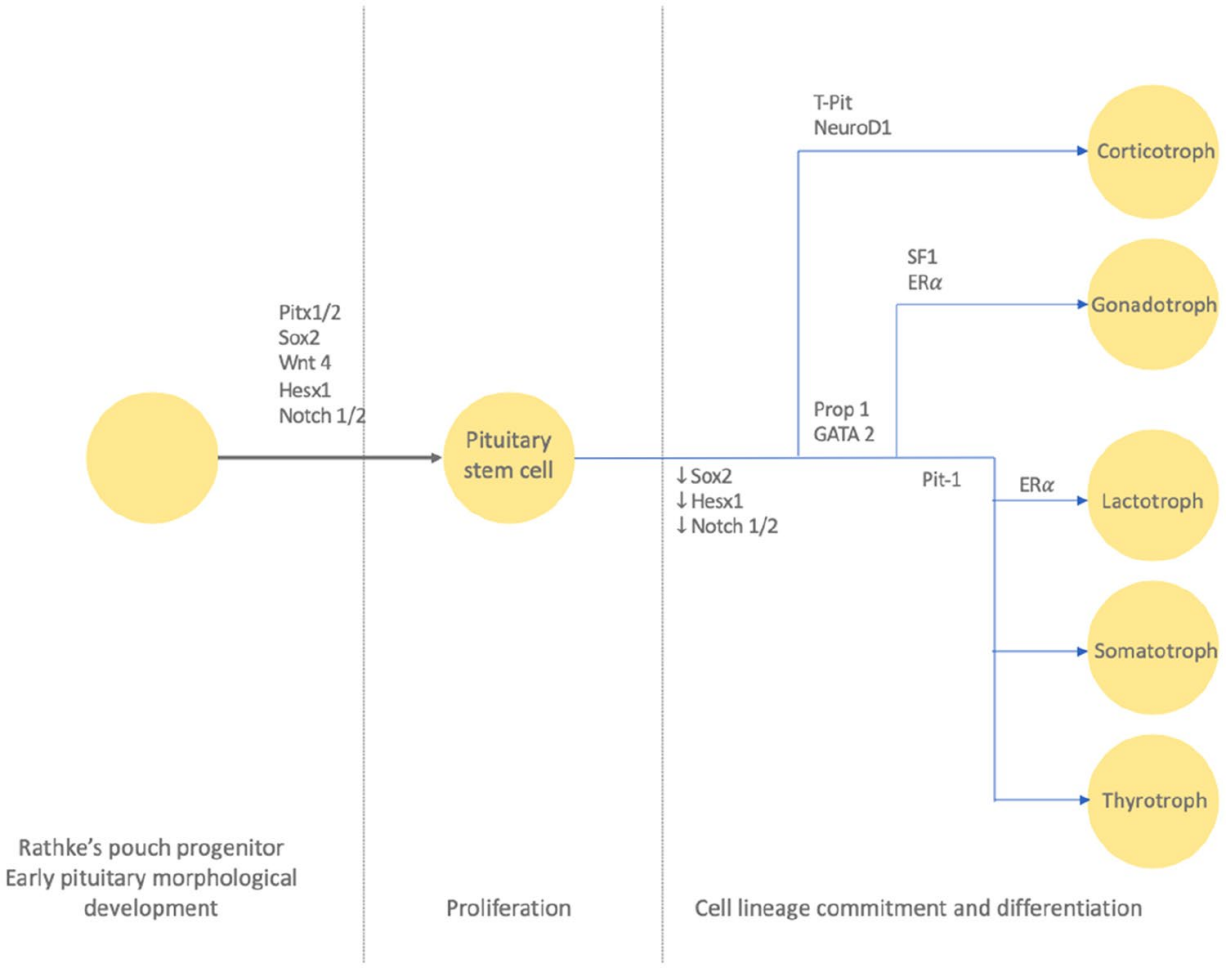
Cell lineage differentiation. Image from Lenders et al. [10]

In 2017, the WHO classification introduced lineage-based classification of pituitary tumours, as determined by transcription factor and hormonal immunohistochemistry (IHC) [1]. Transcription factor analysis, in combination with hormonal IHC provide accurate identification of tumour type. Pituitary stem cells undergo lineage commitment into mature hormone-producing cell types (ACTH, LH, FSH, TSH, GH, PRL) following downregulation of stem cell markers and upregulation of differentiation-specific transcription factors. Steroidogenic factor 1 (SF1) gives rise to gonadotroph cells; T-box transcription factor 19 (TPIT) gives rise to corticotroph cells and pituitary-specific positive transcription factor (PIT1) gives rise to lactotroph, somatotroph and thyrotroph cells (Fig. 1). Adoption of transcription factor analysis has been associated with improved diagnostic and prognostic information, including refinement of classification and improved identification of hormonally silent or ‘whispering’ tumours [2–5]. The recent release of the fifth edition of the WHO classification (2022) builds upon the transcription factor-based classification of pituitary tumours with description of new types, summarised in Table 1 [6].

**Table 1:** Evolution of WHO Classification of pituitary tumours 2004 to 2022

	2004 WHO	2017 WHO		2022 WHO	
IHC	Hormonal	TF and hormonal		TF and hormonal	
Diagnosis	“Typical” adenoma OR “Atypical” adenoma (raised proliferative activity) OR Carcinoma (craniospinal or distant metastases)	*SF1 lineage*	Gonadotroph	*SF1 lineage*	Gonadotroph
		*TPIT lineage*	Corticotroph	*TPIT lineage*	Corticotroph
		PIT1 lineage	Lactotroph (sparsely granulated, densely granulated, ASC)	*Pit1 lineage*	Lactotroph (sparsely granulated, densely granulated)
			Somatotroph (sparsely granulated, densely granulated, mammosomatotroph, mixed somatotroph- lactotroph)		Somatotroph (sparsely granulated, densely granulated)
			Thyrotroph		Mammosomatotroph^c^
			Plurihormonal (PIT1 positive plurihormonal^a^, unusual combinations)		Mixed somatotroph and lactotroph^c^
					Thyrotroph
					Mature plurihormonal PIT1 lineage^b^
					Immature PIT1 lineage^b^
					Acidophil stem cell^c^
		*No distinct cell lineage*	Null cell	*No distinct cell lineage*	Null cell
					Plurihormonal^b^

*IHC* immunohistochemistry, *TF* transcription factor

^a^Newly defined in 2017 WHO Classification

^b^Newly defined in 2022 WHO Classification

^c^Newly described as separate “type” rather than “subtype” in 2022 WHO Classification

However, the expansion of diagnostic IHC is also associated with increased cost and burden of work for laboratory staff [2, 6]. Clinical and economic feasibility pose the need for a tiered approach [6–8]. The European Pituitary Pathology Group (EPPG) proposed a diagnostic algorithm in 2017. The tiered approach commences with clinical evaluation followed by hormonal IHC and cytokeratin staining, with transcription factor IHC in certain situations only, if hormonal IHC was immunonegative, scantly positive or plurihormonal [8]. Authors concede that the algorithm is not suitable for identification of rarer types; moreover it is made less relevant by the 2022 WHO classification, which shifts focus further on transcription factor based classification [6]. Another algorithm was proposed by McDonald et al. in 2017, then revised in 2021 with the addition of TPIT, previously omitted due to lack of commercial availability [7, 9]. Tissue microarrays were created for 136 tumours, with application of a one or two step algorithm: (1) transcription factor IHC and (2) IHC for prolactin, growth hormone, thyroid-stimulating hormone and cytokeratin CAM5.2 for cases that were not clearly gonadotroph, corticotroph or null cell, based on transcription factor IHC alone [7, 9]. Although the algorithm was able to reduce the number of required IHC stains by approximately 30%, there were shortcomings to be addressed. Notably, the algorithm may miss rarer tumours identified within the 2022 WHO classification, such as the “Plurihormonal” tumours with a monomorphous population of cells expressing transcription factors and hormones of multiple lineages.

Herein, we report on our experience in the first two years of clinical application of transcription factor and hormonal IHC for the diagnosis of pituitary tumours in a tertiary referral centre in Australia. We propose a tiered algorithm that builds on that of McDonald et al., utilising transcription factor IHC as the first step in accurate and efficient diagnosis of tumours. We compare this to the current “Gold Standard”, which is clinical evaluation in combination with a full panel of transcription factor and pituitary hormone IHC [2].

## Aim

To determine (1) the utility of the WHO 2022 classification of pituitary tumours in a routine clinical setting, and (2) an optimal algorithm for incorporation of transcription factor IHC in the routine pathological evaluation of pituitary tumour type, improving diagnostic efficacy whilst being cost- and time-efficient for real-world application.

## Methods

This was a retrospective evaluation of transcription factor and hormone IHC performed in routine clinical practice, according to the WHO 2022 classification, on pituitary tumours operated at St Vincent’s Public and Private Hospitals in the years 2019–2021. From 2019 onwards, transcription factor IHC was routinely performed by pituitary pathologists (PE, JL, JC) on all patients with a pituitary tumour operated through St Vincent’s Public and Private Hospitals. IHC was performed using the Ventana Benchmark Ultra automated slide stainer (Ventana Medical Systems, Inc. Tucson, AZ). The following antibodies were used according to protocols established in our laboratory: T-Pit (TBX-19, CL6251; Sigma Aldrich; 1/100), Pit-1 (Pit-1, D7; Santa Cruz; 1/50), SF-1 (SF-1, EPR19744; Abcam; 1/200). Negative and positive controls were run in parallel with each. Transcription factor IHC was considered positive if at least 10% of cells were positive. Other routine histopathological evaluation was performed on all tumour samples, specifically, H&E and reticulin stains to define tumour and normal pituitary, as well as those required for subtype classification according to cell lineage (Cam5.2). Data on patient clinical presentation, pathology and diagnosis were collected.

**Fig. 2:**
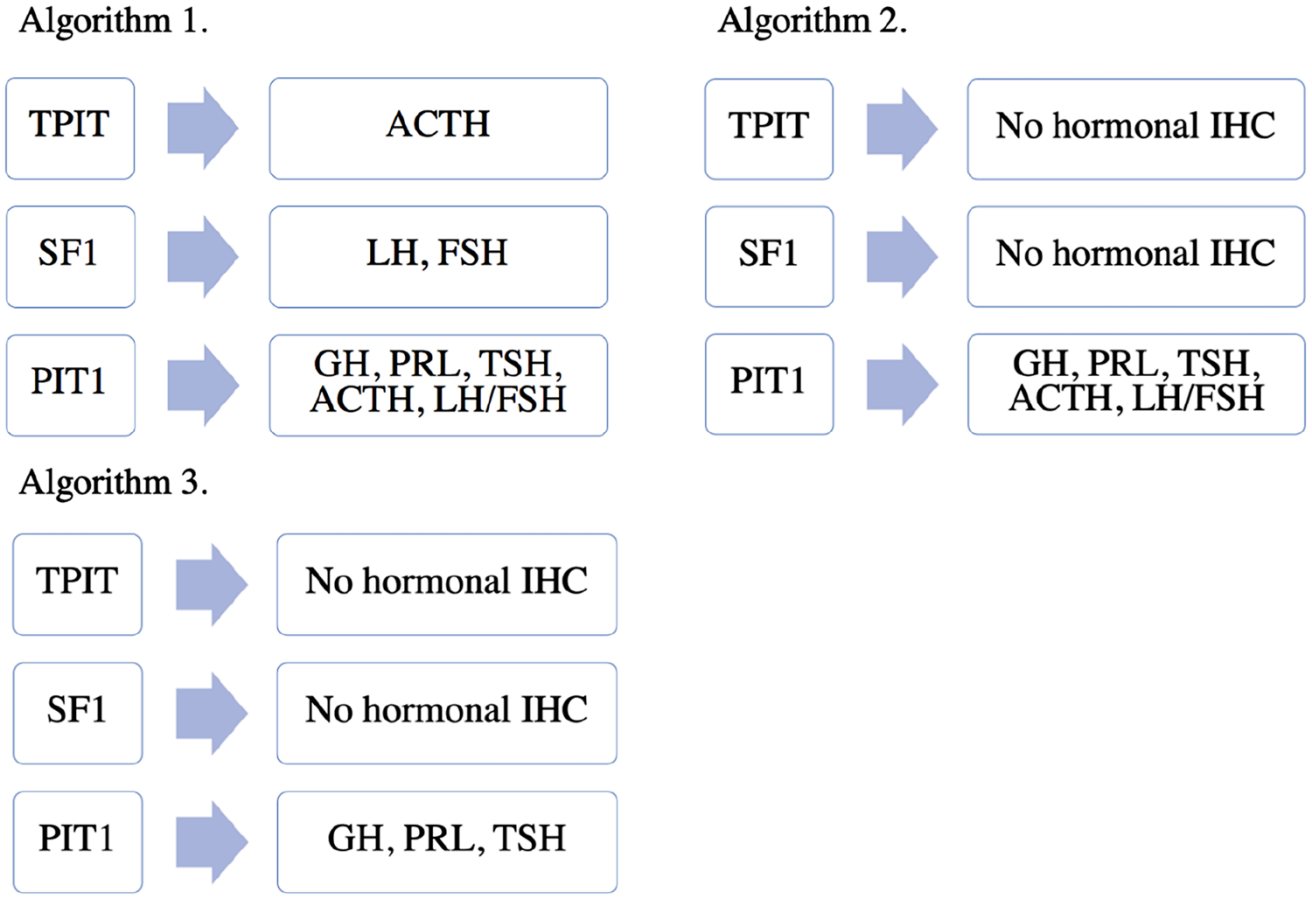
Algorithms tested

Several potential algorithms (Fig. 2) for evaluation of pituitary tumours were investigated. These algorithms were derived by our team of pathologists and endocrinologists, from the WHO 2022 Classification, with a view to developing a tiered approach that would achieve the correct diagnosis, whilst maintaining the most efficient use of IHC [6].

For all patients, a full transcription factor panel was applied first. If more than one transcription factor was positive or if no transcription factors were positive, then a complete hormonal IHC panel was applied. Otherwise, following transcription factor analysis, the tiered algorithms were applied to all other cases. Comparison was made to the “Gold Standard” (current routine practice), comprising clinical evaluation in combination with a full panel of hormonal and transcription factor IHC. Investigators were blinded to the diagnosis based on routine practice when evaluating a diagnosis derived from the algorithms.

Current routine practice for histopathological diagnosis utilises application of 9 immunostains on whole sections of pituitary tumour: PIT1, SF1, TPIT, luteinising hormone (LH), follicle stimulating hormone (FSH), growth hormone (GH), adrenocorticotropic hormone (ACTH), thyroid stimulating hormone (TSH) and prolactin (PRL) with total cost $450 AUD, with additional markers required for refined type diagnosis (low molecular weight keratin, LMWK) and proliferative activity (Ki67). The cost of IHC was estimated to be approximately $50 AUD per immunostain per slide. For each diagnostic algorithm, mean cost per patient was estimated and compared with that of our current routine practice. Ethics approval was obtained from St Vincent’s Hospital.

## Results

Analysis was undertaken in 113 patients with complete whole section hormonal and transcription factor IHC along with clinical data, from a total of 139 pituitary tumours resected between 2019 and 2021. The histological types of included tumours, based on a full panel of transcription factor and hormone IHC analysis, are summarised in Table 2. There were 101 (89.3%) tumours arising from a distinct cell lineage (either PIT1, SF1 or TPIT) with tumours of SF1 lineage forming the larger group (39.8%) followed closely by PIT1 lineage (37.1%) and TPIT (12.4%).

**Table 2:** WHO 2022 ‘Type’ classification of pituitary tumours based on full panel transcription factor and hormonal IHC analysis, 2019–2021, n = 113

Type	Number (%)	Clinically functioning
PIT1 lineage		
Somatotroph tumours	11 (9.7)	10/11
Lactotroph tumours	21 (18.6)	20/21
Mammosomatotroph tumour	3 (2.7)	3/3
Thyrotroph tumour	2 (1.8)	1/2
Mature plurihormonal PIT1 lineage tumour	1 (0.9)	1/1
Immature PIT1 lineage tumour	2 (1.8)	1/2
Acidophil stem cell tumour	0 (0)	0/0
Mixed somatotroph and lactotroph tumour	2 (1.8)	1/2
TPIT lineage		
Corticotroph tumours	14 (12.4)	10/14
SF1 lineage		
Gonadotroph tumour	45 (39.8)	0/45
Tumours with no distinct cell lineage		
Plurihormonal tumour	11 (9.7)	6/11
Null cell tumour	1 (0.9)	0/1

**Fig. 3:**
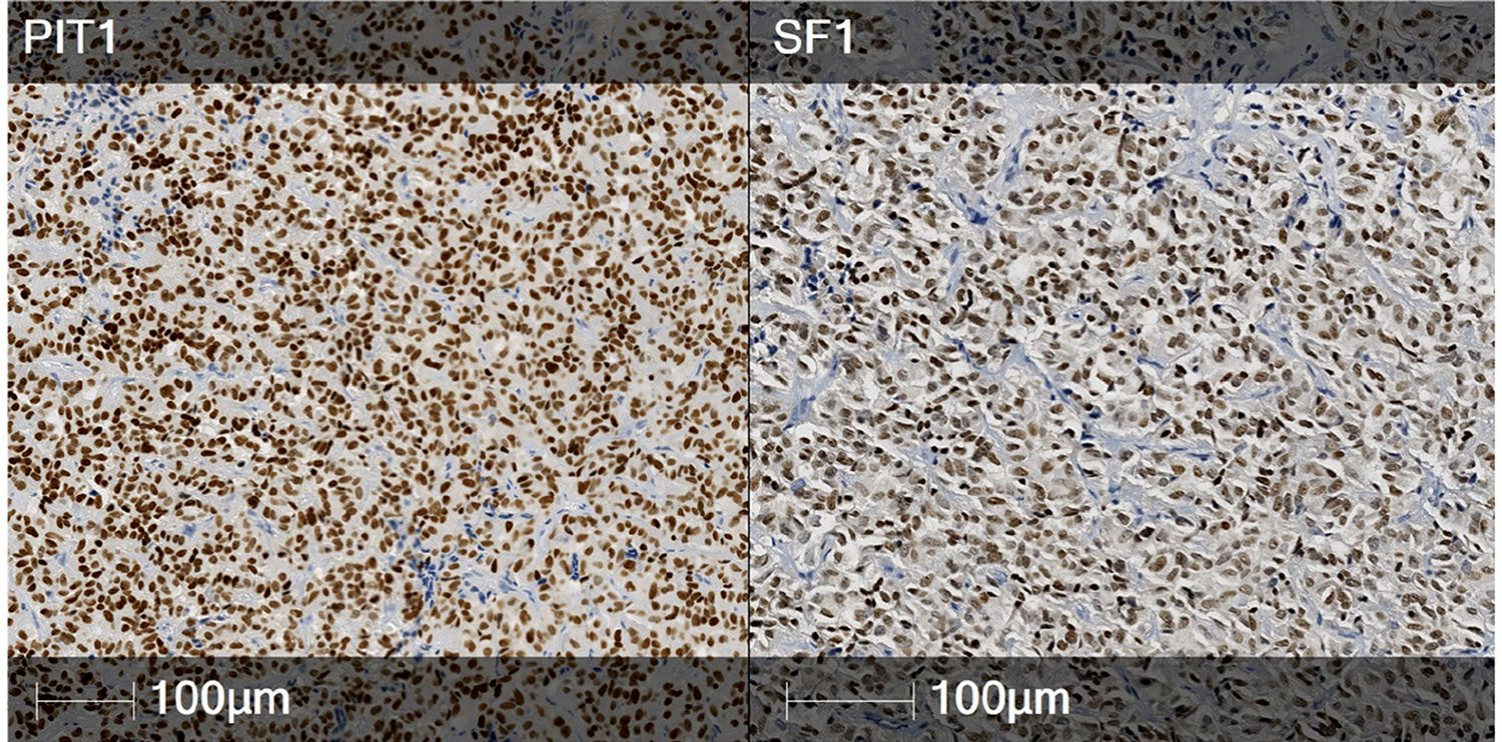
Plurihormonal tumour with no distinct cell lineage: co-expression of PIT1 (left) and SF1 (right)

There were 12 tumours with a “no distinct cell lineage” diagnosis, as per the WHO 2022 classification, summarised in Table 3. There was one null cell tumour presenting as a clinically non-functioning pituitary tumour with no transcription factor or hormonal expression. There were 11 plurihormonal tumours (Fig. 3), with unusual patterns of transcription factor expression, of which 5 were clinically non-functioning, 5 presented with acromegaly and 1 presented with hyperprolactinaemia. Within the PIT1 lineage tumours, co-expression with SF1 was observed in all six cases, with one also expressing TPIT. Hormonal expression was varied, predominantly of PIT1 lineage, with only one case expressing SF1 lineage hormones (FSH). Transcription factor IHC in the clinically non-functioning tumours was more varied, with 2 co-expressing PIT1 and SF1, 2 co-expressing TPIT and SF1 and 1 transcription factor negative. The latter expressed all three PIT1 lineage hormones, whereas the other clinically non-functioning tumours were characterised by scant immunoexpression, with 0 or 1 positive hormones.

**Table 3:** Cases of tumours with no distinct cell lineage (n = 12)

Clinical diagnosis	PRL	TSH	GH	ACTH	LH	FSH	PIT1	SF1	TPIT	WHO 2022 type
Non-functioning	−	−	−	−	−	−	−	−	−	Null cell tumour
Non-functioning	−	−	−	−	−	−	−	+	+	Plurihormonal tumour
Non-functioning	+	+	+	−	−	−	−	−	−	Plurihormonal tumour
Non-functioning	−	−	−	−	−	+	+	+	−	Plurihormonal tumour
Non-functioning	+	−	−	−	−	−	+	+	−	Plurihormonal tumour
Non-functioning	−	−	−	−	−	+	−	+	+	Plurihormonal tumour
Acromegaly	+	+	+	−	−	−	+	+	−	Plurihormonal tumour
Acromegaly	+	+	+	−	−	−	+	+	−	Plurihormonal tumour
Acromegaly	−	−	+	−	−	+	+	+	+	Plurihormonal tumour
Acromegaly	−	+	+	−	−	−	+	+	−	Plurihormonal tumour
Acromegaly	+	+	+	−	−	−	+	+	−	Plurihormonal tumour
Prolactinoma	+	−	−	−	−	−	+	+	−	Plurihormonal tumour

The results of algorithm diagnostic concordance and cost are summarised in Table 4. All algorithms resulted in 100% concordance with “Gold Standard” diagnosis. Each algorithm was associated with reduction in total number of immunostains per patient and also cost, with Algorithm 3 performing the best from a labour and economic perspective.

**Table 4:** Algorithm performance in our cohort (n = 113), compared with full panel of anterior pituitary hormones and transcription factor analysis

Algorithm	Concordance with gold standard (%)	Total number of immunostains applied to cohort (n = 113)	Average number of immunostains applied to cohort (n = 113)	Average cost ($AUD)
1	113 (100)	768	6.8	341
2	113 (100)	667	5.9	296
3	113 (100)	542	4.8	240

## Discussion

Herein we described our clinical experience on the utility of transcription factor analysis in the diagnosis of pituitary tumours, between 2019 and 2021. We have demonstrated the diagnostic breakdown of operated tumours, as per the new 2022 WHO Classification, finding that approximately 10% of tumours present a challenge in accurate classification. In addition, we illustrate that a tiered algorithmic approach achieves excellent diagnostic concordance to a full panel of nine immunostains. This tiered approach offers significant cost reduction and is advantageous in cases with limited diagnostic material.

Tumours with no distinct cell lineage, either expressing nil or multiple transcription factors, were evaluated with a complete panel of hormonal IHC. Recently reclassified within the WHO update, these are now acknowledged as a separate category from other lineages [6]. The newly defined plurihormonal tumour type is described as “very rare”, comprising a monomorphous population of cells that display features of multiple lineages [6]. Strong co-expression of SF1 and PIT1 across a majority of tumour cells, illustrated in this study, excludes the possibility of entrapped normal cells. Null cell tumours are characterised by absence of hormonal and transcription factor immunoexpression [6]. In this study, we report 12 tumours with no distinct cell lineage: 1 null cell and 11 plurihormonal tumours (Table 3). To date, the plurihormonal tumour type has been described in case reports only, and their biology remains incompletely understood [11–13]. One study hypothesised that plurihormonal tumours may arise from immature “stem” cells, though this has not been proven [11]. Further research is required to improve understanding of this newly defined group of tumours.

Several studies have proposed similar diagnostic algorithms [7, 9], confirming comparable results with regards to excellent concordance. Other studies were performed on tissue microarrays rather than whole sections, which were utilised here and are routine clinical practice. All other studies were published prior to the release of the 2022 WHO classification, therefore did not include relevant and important changes [5, 7–9]. The EPPG proposed hormonal IHC in all cases, with subsequent transcription factor IHC in certain circumstances only such as plurihormonal tumours [8]. This approach risks missing tumours with “no distinct cell lineage”, which may express several transcription factors whilst only staining for one hormone, as demonstrated in the present study. Additionally, there is growing appreciation that tumours may demonstrate a spectrum of hormonal expression, meaning that the most consistently sensitive method of diagnosis is through transcription factor analysis [4]. For example, corticotroph tumours may be clinically silent with either TPIT+/ACTH− or TPIT+/ACTH+ immunoexpression, or present with clinically apparent hormone hypersecretion and TPIT+/ACTH+ immunoexpression [4]. In their analysis, McDonald et al. performed transcription factor then hormonal IHC in certain cases. Sufficient consistency within the SF1 and TPIT lineages were observed to suggest that the clinical history along with transcription factor IHC may be adequate for diagnostic purposes [7, 9]. In agreement with this, our Algorithms 2 and 3 omitted LH, FSH and ACTH staining, yet achieved the same diagnosis for all tumours whilst reducing the average number of stains per tumour to 5.9 and 4.8 respectively. Greater heterogeneity has been observed within PIT1 lineage tumours, warranting further hormonal evaluation after transcription factor IHC, in order to clarify diagnosis [7, 9]. In Algorithm 2, a full hormonal IHC panel was applied to tumours that were positive for PIT1, whereas in Algorithm 3 the second step was restricted to hormones derived from PIT1 cell line. Algorithm 2 may capture PIT1 lineage tumours co-expressing unusual combinations of hormones, resulting in identification of plurihormonal tumours with no distinct cell lineage. However, this pattern of IHC is exceedingly rare. We found that this made no difference to the diagnoses attained amongst our tumours, however concede that this may not always be the case, given the heterogeneity generally observed within this group of tumours. Overall, we found Algorithm 3 the best from an economic and efficiency perspective.

Limitations of this study must be acknowledged. The proposed algorithms aim to accurately and economically diagnose pituitary tumour types according to the 2022 WHO Classification. Although routinely performed in our clinical practice, this study does not report on important components of pituitary tumour histopathological evaluation, including H&E, mitotic count, cytokeratin, Ki67 and other IHC results. These factors are used in determining tumour subtype and prognosis, however were not within the scope of this study, in that our aim was to streamline the IHC required for type diagnosis only. Other limitations include relatively small sample size and single centre design. Further large, prospective, multi-centre studies are required to evaluate the clinical utility and optimal implementation of the 2022 WHO Classification of pituitary tumours.

## Conclusions

In a cohort of 113 operatively-managed pituitary tumours, application of 2022 WHO Classification identified 12 cases with no distinct cell lineage, of which 11 were of the newly defined “plurihormonal” type tumours. Tiered algorithmic classification yielded accurate diagnostic typing whilst reducing labour and financial cost. We propose application of Algorithm 3, whereby transcription factors were applied as a screening step, followed by hormonal IHC for PIT1 positive cases and those with no distinct cell lineage. In this cohort, application of Algorithm 3 was associated with cost reduction of approximately 34% when compared with a full panel of hormonal and transcription factor IHC. Whilst cost and workflow efficiency will enable translation of new classification into broad clinical practice, further prospective multicentre studies are required for continued tumour characterisation and optimisation of classification systems.

## Data Availability

Raw data is available upon request.
